# Occupational Noise Exposure and Diabetes Risk

**DOI:** 10.1155/2021/1804616

**Published:** 2021-03-19

**Authors:** Imene Kacem, M. Kahloul, M. Maoua, M. Hafsia, A. Brahem, M. Limam, M. Ghardallou, F. Brahem, H. Aroui, O. El Maalel, H. Kalboussi, S. Chatti, W. Naija, N. Mrizek

**Affiliations:** ^1^Université de Sousse, Faculty of Medicine of Sousse, Sousse, Tunisia; ^2^Department of Occupational Medicine, Farhat Hached Academic Hospital, Sousse, Tunisia; ^3^Department of Anesthesia and Intensive Care, Sahloul Academic Hospital, Sousse, Tunisia; ^4^Department of Occupational Medicine, Sahloul Academic Hospital, Sousse, Tunisia; ^5^Université de Sousse, Faculty of Medicine of Sousse, Department of Family and Community Medicine, Research Laboratory “LR12ES03”, Sousse 4002, Tunisia

## Abstract

**Introduction:**

Noise is one of the most common worldwide environmental pollutants, especially in occupational fields. As a stressor, it affects not only the ear but also the entire body. Its physiological and psychological impacts have been well established in many conditions such as cardiovascular diseases. However, there is a dearth of evidence regarding diabetes risk related to noises.

**Aim:**

To evaluate the relationship between occupational exposure to noise and the risk of developing diabetes.

**Methods:**

This is a cross-sectional analytical study enrolling two groups of 151 workers each. The first group (noise exposed group: EG) included the employees of a Tunisian power plant, who worked during the day shift and had a permanent position. The second group (unexposed to noise group: NEG) included workers assigned to two academic institutions, who were randomly selected in the Occupational Medicine Department of the Farhat Hached University Hospital in Sousse, during periodical fitness to work visits. Both populations (exposed and unexposed) were matched by age and gender. Data collection was based on a preestablished questionnaire, a physical examination, a biological assessment, and a sonometric study.

**Results:**

The mean equivalent continuous sound level was 89 dB for the EG and 44.6 dB for the NEG. Diabetes was diagnosed in 24 workers from EG (15.9%) and 14 workers from NEG (9.3%), with no statistically significant difference (*p*=0.08). After multiple binary logistic regression, including variables of interest, noise did not appear to be associated with diabetes.

**Conclusion:**

Our results did not reveal a higher risk of developing diabetes in workers exposed to noise. Further studies assessing both level and duration of noise exposure are needed before any definitive conclusion.

## 1. Introduction

Diabetes is a major challenging public health priority around the world because of its huge socioeconomic impact [1]. According to the International Diabetes Federation (IFD), it affects actually about 425 million persons worldwide. By 2045, this number may reach 693 million persons [2]. It concerns particularly low- and middle-income countries such as Tunisia, as it had an estimated prevalence of 9.33% in 2014 [1].

Diabetes is also a serious condition because of its frequent complications as well as its high related mortality. In 2017, it was the leading cause of 5 million deaths which corresponds to one death every eight seconds [2]. In addition, it is a common cause of impaired quality of life and health expenditure growth [3, 4].

The diabetes growing incidence all over the world has been attributed to lifestyle changes, particularly with regard to new eating habits, sedentary, and stress [2]. Recently, other modern life changes have been involved such as the work environment. In fact, the increasing progress of technologies and the use of sophisticated machines make noise emerge in the workplace [5]. Considered as the fourth environmental pollutant, noise continues to take alarming dimensions, becoming a real threat especially in the professional environment. In fact, about 250 million workers worldwide are exposed to noise. Exposure to high levels of noise exceeding 85 dB has been reported by 25% of the Tunisian working population [5].

As an environmental stressor, noise has been incriminated in the genesis of several conditions such as hearing loss, sleep disorders, arterial hypertension, myocardial infarction, and cancers [6–9]. It has also been suggested that noise exposure may alter metabolism and increase the risk of obesity [10, 11]. In 2013, Sørensen et al. [12] raised the issue of noise-induced diabetes leading to an increasing number of studies assessing the relationship between noise exposures and developing diabetes [13–15]. Thereby, several hypotheses have been reported suggesting that noise-induced stress leading to an increase in stress hormones levels such as cortisol, and noise-induced sleep disturbances may be potential pathways underlying the association between noise exposure and the development of metabolic disorders, including diabetes [7, 16]. These repercussions should depend on both level and duration of noise exposure. However, published studies focused mainly on air and road traffic noise, which may be less important than the occupational one. In addition, results remain controversial and inconclusive because of heterogeneity in considered methodologies and studied populations.

In this context, this study was conducted to assess the relationship between occupational noise and the risk of developing diabetes.

## 2. Methods

This is a cross-sectional analytical study conducted in the Occupational Medicine Department of Farhat Hached Academic Hospital of Sousse, over a three-month period (from January the 2^nd^, 2017, to March the 31^st^, 2017).

The study population was divided into two groups according to noise exposure. The first group (exposed group: EG) included employees working during the day shift and having a permanent position in the four units (*A*, *B*, *C*, and *D*) of the power plant of the Tunisian Company of Electricity and Gas (TCEG).

The second group (unexposed group: NEG) was randomly enrolled from workers assigned to two academic institutions, during periodical fitness to work visits. Both exposed and unexposed groups were matched by age and gender.

For both groups, inclusion criteria were seniority at the workstation of more than one year, a normal initial physical examination, and metabolic assessment at the prerecruitment medical examination. Subjects with a medical history of endocrinopathy (hypercorticism, acromegaly, and pheochromocytoma), renal failure, and long-term corticosteroid therapy were excluded from the study.

Data collection was based on a preestablished questionnaire (appendix), a physical examination, a biological assessment, and a sonometric study.

The questionnaire explored socioprofessional characteristics, the lifestyle, family and/or personal medical history of diabetes, cardiovascular diseases, and dyslipidemia.

After having explained to each participant the interest of the study, the investigator in charge of the study filled the preestablished questionnaire. Anonymity was respected for all participants. Only participants who agreed to participate in the study were included.

Smoking was defined by a current consumption or a smoking cessation for less than one year. Alcohol consumption and work stress were assessed by binary questions. The energy intake was assessed by converting the daily consumption reported by the employee, using the practitioners' convertor of the interprofessional association of medical centers in the “Ile de France” region [17]. Caloric intake estimation included breakfast, lunch, dinner, and snacks. The energy intake was divided into 3 categories: small eater (<1599 kcal), average eater (between 1600 and 2199 kcal), and large eater (>2200 kcal).

The physical examination involved weight and height measurement. Obesity was assessed according to the WHO classification [18]. Overweight was defined by a body mass index (BMI) ranging between 25 and 29.9 kg/m^2^. Obesity was defined by a BMI ≥ 30 kg/m^2^.

Blood samples were taken in the morning and analyzed in the same laboratory with respect to the same sampling conditions. A period of fasting of at least 12 hours was recommended for the determination of blood glucose.

The WHO definition (2017) has been considered for the diagnosis of diabetes. Thus, diabetes was defined by fasting blood sugar level ≥1.26 g/L (7.00 mmol/L) in two samples. Mild fasting hyperglycemia (MFH) corresponded to a fasting blood sugar level between 1.10 and 1.26 g/L in two samples [19].

Noise mapping was performed using a class 1 sound level meter placed at the ear level of operators. The mapping was based on the identification of noise sources, physical barriers, the company or the academic institution plan, and the extent location of each workstation. The calibration of the sound level meter was performed according to the manufacturer's instructions. Results were expressed in A-weighted decibels (dB (A)).

Data were analyzed using the software SPSS 18.0. The normality of distribution of continuous variables was tested by a one-sample Kolmogorov–Smirnov test. Continuous variables with normal distribution were presented as means ± standard deviations. Nonnormally distributed variables were reported as medians and interquartile ranges. Categorical variables were expressed as numbers and percentages. For the comparison of means, Student's “*t*” test and Mann–Whitney *U* test were used. Frequencies were compared by the Pearson Chi-square test. Multiple binary logistic regression was performed for multivariate analysis. For all statistical tests, the significance level *p* was set at 0.05.

Subsequently, multivariate analyzes were carried out according to the step-by-step descending multiple binary logistic regression method. The dependent variable was diabetes and the explanatory variables were all variables with *p* less than or equal to 20% in the unvaried analysis.

## 3. Results

**Figure 1:**
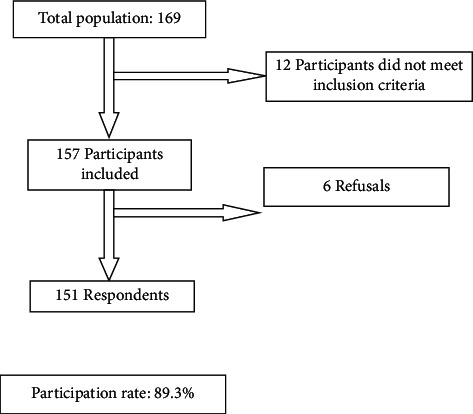
Flow diagram.

Both groups included 151 employees (Figure 1). The median age of the study population was 44 years with extremes ranging from 25 to 60 years and markedly male dominance (85.4%). Comparisons between both groups according to sociooccupational characteristics and lifestyle are shown in Table 1.

Tobacco consumption was reported by 54.7% in the EG versus 44.2% in NEG without a statistically significant difference (*p*=0.07). Physical activity practice was reported by 29.1% in the EG versus 41.7% in the NEG, with a statistically significant difference (*p*=0.03).

The technician position was most frequently held by the EG workers (39.1%) and the position of Administrative Officer was most frequently occupied by workers in the NEG (21.8%).

The median job seniority in EG was 6 years with extremes ranging from 1 to 38 years with a significantly higher median (*p*=0.02) among NEG (12 years; [2–38 years]). Work related stress was reported by 57% of workers in the EG and 18.5% of workers in the NEG, with a statistically significant difference (*p*<10^−3^).

The mean equivalent continuous sound level was 89 dB for the exposed group and 44.6 dB for the unexposed group. The highest sound levels in the EG were noted at the gas turbine of the plant *B* = 103 dB (A), gas turbine of the plant *C* = 100 dB (A), seawater desalination station of the plant *D* = 93 dB (A), and steam turbine combined cycle of the plant *B* = 92 dB (A).

According to the energy intake, large eaters were found in 17.9% of cases in EG and 55% of cases in NEG with a statistically significant difference (*p*<10^−3^) (Table 1). Hypertension was the most frequently reported disease in the family history of our study population (51% in EG and 36.4% in NEG).

Diabetes was diagnosed in 24 noise exposed employees (15.9%) and 14 unexposed employees (9.3%), with no statistically significant difference (*p*=0.14).  Table 2 shows the distribution of workers according to clinical and biological parameters.

After bivariate analysis, diabetes was associated with age, job seniority, physical exercise, energy intake, and stress (Table 3).

After multiple binary logistic regressions, factors independently associated with diabetes were physical activity (ORa = 0.37), a high daily calorie intake (ORa = 3.15), job seniority (ORa = 1.015), and stress (ORa = 3.201). Occupational noise exposure did not seem to be associated with diabetes (Table 4).

## 4. Discussion

Occupational noise was not found to be associated with diabetes either in our study or in the literature [20, 21]. However, a higher risk of diabetes was attributed to other types of noise exposure [22–26]. In addition, noise exposure consequences have been involved in many conditions that have similar pathogenesis with diabetes. Thus, it still seems difficult to make a definitive conclusion before conducting a large study taking into consideration a rigorous analysis of published data.

In the cross-sectional study of Dzhambov [20] enrolling 28.221 participants from 15 European countries, noise exposure in the workplace was not associated with a higher risk of diabetes (OR = 1.01, 95% CI: 0.78–1.32). However, an increased risk was reported in patients aged above 65 years (9% (95% CI: −9–31%) and men (12% (95% CI: −13–45%)). In addition, noise exposure and diabetes assessments were based only on binary questions: “Have you been exposed?” “Are you being followed for diabetes?”

Similar results were reported by Song [21] in his National Population Health Survey (NPHS) investigating the effect of occupational noise exposure on the risk of diabetes, rheumatoid arthritis, and cardiovascular disease. The exposure assessment was performed by constructing a work-related noise exposure matrix, assigning the exposure level according to the job titles of the subjects, and cumulating the exposure on the duration declared by the jobs.

Zare Sakhvidi et al. [14] reported other arguments that could explain the absence of a relationship between occupational noise exposure and the risk of diabetes mellitus. In fact, the use of protective equipment, higher physical activity, and the good health being status of workers (“healthy worker effect”) may minimize noise exposure consequences in the workplace, with comparison to other types of noise exposure such as road traffic noise.

According to a Danish study enrolling 50.187 adults, a 10 dB higher level of exposure to road traffic noise at the current residence and during the previous 5 years was associated with statistically significant 8% (95% CI: 1.02, 1.14) and 11% (95% CI: 1.05, 1.18) higher risk of incident diabetes, respectively [12].

Clark et al. [22] assessed the impact of traffic noise exposure on the incidence of diabetes over a 5-year period. This study enrolled 380.738 persons aged between 45 and 85 years and exposed to an average noise level of 63 dB. An increase of 8% in the incidence of diabetes was found.

Similar results have been reported by another study conducted in Switzerland between 2002 and 2011 and enrolling 2.631 participants exposed to two different average levels of noise: 54 dB for road traffic noise and 30 dB for aircraft noise [23]. Noise exposure was significantly associated with diabetes occurrence with a RR of 1.35 (95% CI: 1.02) and 1.86 (95% CI: 0.96–3.59), respectively.

Dzhambov and Dimitrova1 [24] reported a significantly higher risk of diabetes type 2 in persons exposed to road traffic noise of 71 to 80 dB with a relative risk of 4.49 (95% CI: 1.38 to 14.68).

Similar results were found in patients living close to noisy roads in comparison to those living close to quiet ones. A higher risk of developing diabetes was attributed to very noisy roads (OR 1.49, 95% CI: 1.04–2.14) and extremely noisy roads (OR 1.99, 95% CI: 1.14–3.47). After adjusting for confounding variables, the risk persisted only for extremely noisy roads (OR: 1.97, 95% CI: 1.07–3.64) [25]. A recent study assessing the extra-auditory effects of noise in 1836 participants in South Korea concluded a significantly higher risk of diabetes with exposure to high levels of noise (OR 1.5, 95% CI 1.04–2.25) (*p*=0.028) [26].

Another meta-analysis published in 2015 including 9 studies (5 case-control and 4 cohorts) reported a higher risk of diabetes mellitus ([22% (95% CI: 9%, 37%)) in subjects exposed to noise greater than 64 dB compared to those with noise exposure <64 dB [15].

Finally, a recent meta-analysis including 15 articles related to the relationship between noise exposure and diabetes (6 cohorts, 6 cross sections, and 3 case-controls) found a 6% increase in the risk of diabetes mellitus associated with a 5 dB increase in noise exposure [14]. The authors also reported stronger associations for air traffic followed by road traffic. In fact, for an increase in noise exposure of 5 dB, there was a 17% increase in the risk for air traffic noise and 7% for road traffic. However, rail traffic noise has not been associated with an increased risk of diabetes mellitus.

The impact of exposure noise on diabetes has been explained by the interaction of two main pathways which are stress and sleep disturbances [7]. As a stress inducer, noise can increase catecholamine synthesis, inducing insulin resistance and glucose homeostasis disorders [26, 27]. These disorders are likely to be accentuated by sleep disturbances related to noise [28] that can result in both altered regulation of blood glucose and increased adiposity [29]. Thus, exposure to high-intensity noise interferes with several physiological, metabolic, and immunological functions [30, 31].

The perception of noise, which is a loud or unpleasant sound, is a complex process involving two auditory pathways, one from the inner ear to the auditory cortex and the other from the inner ear to the reticular activating system. Connected to the limbic system as well as the autonomic nervous system and the neuroendocrine system, the activation of these pathways interferes with the synthesis of adrenaline, noradrenaline, and corticosteroids [32, 33]. Effects of noise on the sympathetic branch of the autonomic nervous system and on the hypothalamic-pituitary-adrenal axis are supported by observational data [34] and experimental results [35]. In addition, long-term exposure to noise has been suggested to cause an imbalance in the mechanism of stress regulation, increasing the risk of cardiovascular disease [36]. Chronic increase of stress hormone levels induces hypertonic and diabetogenic effects and may lead to alterations in adipose tissue metabolism [37]. Thus, such a chronic state of stress may contribute to the development of obesity, insulin resistance, and type 2 diabetes [38, 39]. Furthermore, high levels of cortisol are associated with insulin resistance and/or hyperinsulinemia, including increased gluconeogenesis; visceral fat cell growth, carbohydrate intolerance; increased total cholesterol, LDL, and triglycerides; decreased HDL, and impaired insulin secretion [40].

In addition, noise is generally associated with sleep disturbances. Chronic sleep deficiency [41] can alter a person's well-being, metabolism, and endocrine function [42]. Sleep debt has been shown to be responsible for disrupting carbohydrate metabolism by reducing glucose tolerance and increasing sympathetic nervous system activity [43]. Shortening of sleep may also affect serum levels of leptin and ghrelin, resulting in increased appetite and reduced energy expenditure, increasing the risk of overweight and obesity [44]. According to a recent systematic review and meta-analysis, reduced and impaired sleep quality could predict the risk of developing type 2 diabetes [29].

Although our study has the originality of assessing objectively the levels of noise exposures in exposed and nonexposed individuals, some limitations should be considered.

In fact, the sample size was relatively small despite a participation rate of around 89.3%. Thus, the extrapolation of the results is quite difficult.

In addition, a selection bias due to the “healthy worker” effect should be considered. In fact, in cross-sectional surveys, employees who stopped working or were not fit for work were not enrolled in the study. The risk is therefore an underestimation of the prevalence of diabetes. However, despite these limitations, our population was homogeneous as the workers belonged to the same company and had the same constraints.

On the other hand, although the cross-sectional nature of the study has the advantage of being more practical, with low cost and short duration, it does not make it possible to establish causal links between the studied variables. In addition, the use of self-questionnaire in the collection of data exposes to a bias of subjectivity for some parameters especially those related to psychological stress. However, the list of studied variables was quite exhaustive and framed with the set of potential risk factors involved in the genesis of diabetes, and data collection and clinical examination were performed by the same doctor, in order to reduce the measurement bias.

Finally, the strength of our study was the sonometric evaluation which made it possible to establish a mapping in order to appreciate the distribution of sound levels in the different local of the study.

## 5. Conclusion

Our results did not reveal a higher risk of diabetes related to noise. Further studies assessing both level and duration of occupational noise exposure are needed before any definitive conclusion. Meanwhile, the established consequences of noise justify effective workplace interventions in the power plant.

## Figures and Tables

**Table 1:** Socioprofessional characteristics and lifestyle habits of the study population.

Variables	Exposed group		Unexposed group		*p*
	Number	%	Number	%	
*Level of study*					
Primary	10	6.6	27	17.9	**0.01**
Secondary	72	47.7	64	42.4	
University	69	45.7	60	39.7	

*Marital status*					
Married	105	69.5	105	69.5	1
Others	46	30.5	46	30.5	

*Number of children*					
≤2	108	71.5	104	68.9	0.61
>2	43	28.5	47	31.1	

*Tobacco smoking*					
Yes	82	54.7	65	44.2	**0.07**
No	68	45.3	82	55.9	

*Alcohol consumption*					
Yes	16	10.6	15	9.9	0.85
No	135	89.4	136	90.1	

*Physical activity*					
Yes	44	29.1	62	41.1	**0.03**
No	107	70.9	89	58.9	

*Leisure activity*					
Yes	35	23.2	19	12.6	0.16
No	116	76.8	132	87.4	

*Energy intake*					
Small eater	49	32.5	10	10	**<10** ^**−3**^
Average eater	75	49.7	58	38.4	
Large eater	27	17.9	83	55	

*Occupational status*					
Technician	41	27.2	29	19.2	^*∗*^
Worker	59	39.1	17	11.3	
Engineer	26	17.2	6	4	
Administrative agent	21	13.8	33	21.8	
Security agent	1	0.7	13	8.7	
Cleaner	0	0	10	6.6	

*Perceived stress*					
Yes	86	57	28	18.5	**<10** ^**−3**^
No	65	43	123	81.5	

^*∗*^Not applicable test.

**Table 2:** Clinical and biological parameters of the study population.

Variables	Exposed group		Unexposed group		*p*
	Number	%	Number	%	
*Family history*					
Diabetes	73	48.3	37	24.5	**<10** ^**−3**^
HBP	77	51	55	36.4	**0.01**
Stroke	6	4	13	8.6	0.09

*Personal history*					
Diabetes	10	6.6	6	4	0.30
HBP	12	7.9	10	6.6	0.65

*Diabetes*					
No	107	70.9	121	80.1	0.14
MFH	20	13.2	16	10.6	
Yes	24	15.9	14	9.3	

	Mean (SD)		Mean (SD)		
	^*∗*^Median (min-max)		^*∗*^Median (min-max)		
BMI	29.3 (3.5) kg/m^2^		28.2 (4.8) kg/m^2^		**0.02**

Fasting blood sugar level	5.35 [2.33–12.22] mmol/L		5.13 [4.06–15.5] mmol/L		**0.03**

HBP: high blood pressure; SD: standard deviation; min: minimum; max: maximum; BMI: body mass index MFH: mild fasting hyperglycemia; ^*∗*^not applicable test.

**Table 3:** Associated factors to diabetes at the univariate analysis.

Variables	Diabetes				*p*
	Yes		No		
	N	%	N	%	
*Gender*					
Man	34	13.2	224	86.8	0.45
Woman	4	9.1	40	90.9	

*Marital status*					
Married	28	73.3	182	68.9	0.55
Others	10	26.3	82	31.1	

*Level of study*					
Primary	2	5.3	35	13.3	^*∗*^
Secondary	24	63.2	112	42.4	
University	12	31.6	117	44.3	

*Number of children*					
≤2	23	60.5	189	71.6	0.16
>2	15	39.5	75	28.4	

*Tobacco consumption*					
Yes	21	55.3	126	48.6	0.44
No	17	44.7	133	51.4	

*Alcohol consumption*					
Yes	5	13.2	33	86.8	0.73
No	33	86.8	238	90.2	

*Physical activity*					
Yes	7	18.4	99	37.5	**0.02**
No	31	81.6	165	62.5	

*Leisure activity*					
Yes	4	10.5	50	18.9	0.20
No	34	89.5	214	81.1	

*Energy intake*					
Small and average eater	17	44.7	175	66.3	**0.01**
Large eater	21	55.3	55.3	33.7	

*Obesity*					
Yes	21	55.3	104	39.4	0.06
No	17	44.7	160	60.6	

*Stress*					
Yes	22	57.9	92	34.8	**0.00**
No	16	42.1	172	65.2	

*Family history of diabetes*					
Yes	19	50	91	34.5	0.06
No	19	50	173	65.5	

*Noise*					
Yes	24	63.2	127	48.1	0.08
No	14	36.8	137	51.9	

	Mean ± SD		Mean ± SD		
	^*∗*^Median (min-max)		^*∗*^Median (min-max)		
Age (years)	46 [26–60]		42 [25–60]		**0.03**
Job seniority (years)	19 [2–37]		10 [1–38]		**0.00**

^*∗*^Not applicable test; SD: standard deviation.

**Table 4:** : Associated factors to diabetes in multivariate analysis.

Variables	*p*	ORa	CI 95%
Physical activity	0.03	0.37	0.14–0.91
Energy intake (large eater)	0.00	3.15	1.4–6.66
Job seniority	0.00	1.05	1.01–1.08
Stress	0.00	3.20	1.52–6.72

ORa: adjusted odds ratio; CI: confident interval variables adjusting the model: number of children, leisure activity, family history of diabetes, age, noise.

## Data Availability

The data used to support the findings of this study are available upon request to the corresponding author via email.

## Supplementary Materials

Supplementary Materials The survey considered in this study has been attached as a supplementary file.
