# Supplementary material for: Occupational Noise Exposure and Diabetes Risk
Source: J Environ Public Health. 2021 Mar 19;2021:1804616. doi: 10.1155/2021/1804616 (PMC8004364; doi:10.1155/2021/1804616)
Supplement: Supplementary Materials — The survey considered in this study has been attached as a supplementary file. [file 1804616.f1.docx]

**Appendix**

**Occupational noise exposure and diabetes risk**

***Socio-demographic characteristics:***

1. Gender: 0 = male I__I

1 = female

1. Date of birth : I I I I I
2. Marital status :0 = Married I__I

1= Others

1. Number of children :

0= 0 child I__I

1= 1 child

2= 2 children

3= 3 children

4≥ 4 children

1. Level study 0= Primary I__I

1= Secondary

2= University

***Lifestyle habits :***

1. Physical activiy ? 0=No I__I

1= Yes

1. Leisure Activity ?  :

0=No I__I

1= Gardening, 2= Music

3= Sewing 4= Others : Specify :……………………….

1. Tobacco smoking : 0= No I__I

1= Yes

1. Type : 1= Cigarette

2=Hookah

Numbers of cigarette/ day :……………………

Numbers of Hookah/ week : ……………….

1. If weaned since when …………..
2. Alcohol consumption: 0= No I__I

1= Yes

1. Type of drink :…………………
2. Numbers of drinks/ day : …………………
3. Means of transport  : 1= Car I__I

2 = feet

3= Bus

4= Taxi

5= Motocycle/bicycle

1. Usual day’s diet :

Breakfest:………………………………………………………………………… Lunch:…………………………………………………………………………………

Dinner :…………………………………………………………………………………… Snacks:…………………………………………………………………………….

***Professional characteristics:***

1. Occupational status

1= Worker I__I

2= Cleaner

3= Technician

4= Engineer

5= Administrative agent

6= Security agent

7= Others

Specify…………………………..

1. Job seniority (years) : I__I__I
2. Stress in work : 0 = No I__I

1= Yes

***Medical history :***

1. Family history

1= No

2=Diabetes

3= HBP

4= Stroke

5= Others

Specify  :………………………………………

1. Personal history after recrutement :

1= No

2=Diabetes

3= HBP

4= Stroke

5= Dyslipidemia

6= Dysthyroidism

7= Others

Specify ………………………………………….

***Physical examination***

1. Weight I__I__I
2. Seize I__I__I
3. Blood pressure 1 measurement : I__I__I ; I__I__I

2 measurement : I__I__I ; I__I__I

**Biological assensement:**

1. Fasting blood sugar level :………………….
